# Supplementary material for: Pyruvate Dehydrogenase Kinase 1 inhibition mediated oxidative phosphorylation enhancement in cartilage promotes osteoarthritis progression
Source: BMC Musculoskelet Disord. 2023 Jul 20;24:597. doi: 10.1186/s12891-023-06585-6 (PMC10357736; doi:10.1186/s12891-023-06585-6)
Supplement: Supplementary file 1 — Additional file 1. [file 12891_2023_6585_MOESM1_ESM.zip › MF.Gsea.1653627316242/GOMF_GTPASE_BINDING.html]

Details for gene set GOMF\_GTPASE\_BINDING[GSEA]

|  || Dataset | 55457\_collapsed\_to\_symbols.cls.cls #OA\_versus\_normal.cls.cls #OA\_versus\_normal\_repos |
| Phenotype | cls.cls#OA\_versus\_normal\_repos |
| Upregulated in class | normal |
| GeneSet | GOMF\_GTPASE\_BINDING |
| Enrichment Score (ES) | -0.3340347 |
| Normalized Enrichment Score (NES) | -1.6279033 |
| Nominal p-value | 0.0 |
| FDR q-value | 0.14419363 |
| FWER p-Value | 0.985 |
Table: GSEA Results Summary

  

Fig 1: Enrichment plot: GOMF\_GTPASE\_BINDING      
 Profile of the Running ES Score & Positions of GeneSet Members on the Rank Ordered List

  

| SYMBOL | TITLE | RANK IN GENE LIST | RANK METRIC SCORE | RUNNING ES | CORE ENRICHMENT || 1 | RALBP1 | ralA binding protein 1 [Source:HGNC Symbol;Acc:HGNC:9841] | 217 | 0.808 | -0.0068 | No |
| 2 | RAB8A | "RAB8A, member RAS oncogene family [Source:HGNC Symbol;Acc:HGNC:7007]" | 383 | 0.712 | -0.0107 | No |
| 3 | CIB1 | calcium and integrin binding 1 [Source:HGNC Symbol;Acc:HGNC:16920] | 436 | 0.691 | -0.0060 | No |
| 4 | XPO7 | exportin 7 [Source:HGNC Symbol;Acc:HGNC:14108] | 443 | 0.686 | 0.0023 | No |
| 5 | DENND1B | DENN domain containing 1B [Source:HGNC Symbol;Acc:HGNC:28404] | 450 | 0.683 | 0.0105 | No |
| 6 | YIPF2 | Yip1 domain family member 2 [Source:HGNC Symbol;Acc:HGNC:28476] | 517 | 0.654 | 0.0137 | No |
| 7 | EPRS1 | glutamyl-prolyl-tRNA synthetase 1 [Source:HGNC Symbol;Acc:HGNC:3418] | 581 | 0.629 | 0.0167 | No |
| 8 | RANGRF | RAN guanine nucleotide release factor [Source:HGNC Symbol;Acc:HGNC:17679] | 660 | 0.603 | 0.0183 | No |
| 9 | PDE6D | phosphodiesterase 6D [Source:HGNC Symbol;Acc:HGNC:8788] | 718 | 0.589 | 0.0213 | No |
| 10 | RPH3AL | rabphilin 3A like (without C2 domains) [Source:HGNC Symbol;Acc:HGNC:10296] | 816 | 0.565 | 0.0209 | No |
| 11 | FGD6 | "FYVE, RhoGEF and PH domain containing 6 [Source:HGNC Symbol;Acc:HGNC:21740]" | 930 | 0.532 | 0.0187 | No |
| 12 | RRAGB | Ras related GTP binding B [Source:HGNC Symbol;Acc:HGNC:19901] | 953 | 0.528 | 0.0237 | No |
| 13 | GOLGA5 | golgin A5 [Source:HGNC Symbol;Acc:HGNC:4428] | 1016 | 0.514 | 0.0254 | No |
| 14 | RIMS3 | regulating synaptic membrane exocytosis 3 [Source:HGNC Symbol;Acc:HGNC:21292] | 1022 | 0.512 | 0.0315 | No |
| 15 | RIMS2 | regulating synaptic membrane exocytosis 2 [Source:HGNC Symbol;Acc:HGNC:17283] | 1159 | 0.485 | 0.0270 | No |
| 16 | RRAGD | Ras related GTP binding D [Source:HGNC Symbol;Acc:HGNC:19903] | 1186 | 0.479 | 0.0311 | No |
| 17 | PLCE1 | phospholipase C epsilon 1 [Source:HGNC Symbol;Acc:HGNC:17175] | 1505 | 0.428 | 0.0114 | No |
| 18 | GNB2 | G protein subunit beta 2 [Source:HGNC Symbol;Acc:HGNC:4398] | 1550 | 0.420 | 0.0133 | No |
| 19 | CYFIP2 | cytoplasmic FMR1 interacting protein 2 [Source:HGNC Symbol;Acc:HGNC:13760] | 1636 | 0.408 | 0.0118 | No |
| 20 | RAB3GAP1 | RAB3 GTPase activating protein catalytic subunit 1 [Source:HGNC Symbol;Acc:HGNC:17063] | 1766 | 0.389 | 0.0066 | No |
| 21 | STOML2 | stomatin like 2 [Source:HGNC Symbol;Acc:HGNC:14559] | 1819 | 0.382 | 0.0074 | No |
| 22 | PFN1 | profilin 1 [Source:HGNC Symbol;Acc:HGNC:8881] | 1863 | 0.377 | 0.0088 | No |
| 23 | ATP6AP1 | ATPase H+ transporting accessory protein 1 [Source:HGNC Symbol;Acc:HGNC:868] | 1866 | 0.377 | 0.0135 | No |
| 24 | USP6NL | USP6 N-terminal like [Source:HGNC Symbol;Acc:HGNC:16858] | 1999 | 0.361 | 0.0076 | No |
| 25 | CYRIA | CYFIP related Rac1 interactor A [Source:HGNC Symbol;Acc:HGNC:25373] | 2102 | 0.349 | 0.0041 | No |
| 26 | HPS6 | HPS6 biogenesis of lysosomal organelles complex 2 subunit 3 [Source:HGNC Symbol;Acc:HGNC:18817] | 2131 | 0.346 | 0.0063 | No |
| 27 | MARCHF5 | membrane associated ring-CH-type finger 5 [Source:HGNC Symbol;Acc:HGNC:26025] | 2150 | 0.344 | 0.0092 | No |
| 28 | EXOC2 | exocyst complex component 2 [Source:HGNC Symbol;Acc:HGNC:24968] | 2230 | 0.336 | 0.0073 | No |
| 29 | EXOC5 | exocyst complex component 5 [Source:HGNC Symbol;Acc:HGNC:10696] | 2243 | 0.334 | 0.0106 | No |
| 30 | RABGAP1L | RAB GTPase activating protein 1 like [Source:HGNC Symbol;Acc:HGNC:24663] | 2334 | 0.322 | 0.0076 | No |
| 31 | DAAM1 | dishevelled associated activator of morphogenesis 1 [Source:HGNC Symbol;Acc:HGNC:18142] | 2402 | 0.314 | 0.0063 | No |
| 32 | SOD1 | superoxide dismutase 1 [Source:HGNC Symbol;Acc:HGNC:11179] | 2413 | 0.313 | 0.0096 | No |
| 33 | XPO1 | exportin 1 [Source:HGNC Symbol;Acc:HGNC:12825] | 2477 | 0.305 | 0.0085 | No |
| 34 | SPHK2 | sphingosine kinase 2 [Source:HGNC Symbol;Acc:HGNC:18859] | 2478 | 0.305 | 0.0124 | No |
| 35 | TNFAIP1 | TNF alpha induced protein 1 [Source:HGNC Symbol;Acc:HGNC:11894] | 2499 | 0.303 | 0.0147 | No |
| 36 | RASA1 | RAS p21 protein activator 1 [Source:HGNC Symbol;Acc:HGNC:9871] | 2506 | 0.302 | 0.0181 | No |
| 37 | PKN1 | protein kinase N1 [Source:HGNC Symbol;Acc:HGNC:9405] | 2540 | 0.298 | 0.0193 | No |
| 38 | RHOH | ras homolog family member H [Source:HGNC Symbol;Acc:HGNC:686] | 2680 | 0.282 | 0.0119 | No |
| 39 | DVL2 | dishevelled segment polarity protein 2 [Source:HGNC Symbol;Acc:HGNC:3086] | 2693 | 0.280 | 0.0145 | No |
| 40 | MLPH | melanophilin [Source:HGNC Symbol;Acc:HGNC:29643] | 2794 | 0.270 | 0.0101 | No |
| 41 | UNC13B | unc-13 homolog B [Source:HGNC Symbol;Acc:HGNC:12566] | 2820 | 0.268 | 0.0115 | No |
| 42 | RNF41 | ring finger protein 41 [Source:HGNC Symbol;Acc:HGNC:18401] | 3005 | 0.249 | 0.0002 | No |
| 43 | RAB29 | "RAB29, member RAS oncogene family [Source:HGNC Symbol;Acc:HGNC:9789]" | 3020 | 0.247 | 0.0022 | No |
| 44 | RANBP1 | RAN binding protein 1 [Source:HGNC Symbol;Acc:HGNC:9847] | 3214 | 0.229 | -0.0101 | No |
| 45 | CYFIP1 | cytoplasmic FMR1 interacting protein 1 [Source:HGNC Symbol;Acc:HGNC:13759] | 3277 | 0.223 | -0.0121 | No |
| 46 | MYO5A | myosin VA [Source:HGNC Symbol;Acc:HGNC:7602] | 3330 | 0.218 | -0.0135 | No |
| 47 | IFT20 | intraflagellar transport 20 [Source:HGNC Symbol;Acc:HGNC:30989] | 3403 | 0.211 | -0.0165 | No |
| 48 | RABGGTA | Rab geranylgeranyltransferase subunit alpha [Source:HGNC Symbol;Acc:HGNC:9795] | 3491 | 0.203 | -0.0207 | No |
| 49 | MYRIP | myosin VIIA and Rab interacting protein [Source:HGNC Symbol;Acc:HGNC:19156] | 3573 | 0.196 | -0.0246 | No |
| 50 | STK19 | serine/threonine kinase 19 [Source:HGNC Symbol;Acc:HGNC:11398] | 3585 | 0.195 | -0.0230 | No |
| 51 | MYO1C | myosin IC [Source:HGNC Symbol;Acc:HGNC:7597] | 3730 | 0.183 | -0.0320 | No |
| 52 | YIPF1 | Yip1 domain family member 1 [Source:HGNC Symbol;Acc:HGNC:25231] | 3774 | 0.179 | -0.0331 | No |
| 53 | GNB3 | G protein subunit beta 3 [Source:HGNC Symbol;Acc:HGNC:4400] | 3800 | 0.177 | -0.0329 | No |
| 54 | PAK1 | p21 (RAC1) activated kinase 1 [Source:HGNC Symbol;Acc:HGNC:8590] | 3861 | 0.169 | -0.0354 | No |
| 55 | GNB1 | G protein subunit beta 1 [Source:HGNC Symbol;Acc:HGNC:4396] | 3969 | 0.158 | -0.0419 | No |
| 56 | ARHGDIB | Rho GDP dissociation inhibitor beta [Source:HGNC Symbol;Acc:HGNC:679] | 4085 | 0.147 | -0.0491 | No |
| 57 | CORO1C | coronin 1C [Source:HGNC Symbol;Acc:HGNC:2254] | 4132 | 0.143 | -0.0509 | No |
| 58 | NGFR | nerve growth factor receptor [Source:HGNC Symbol;Acc:HGNC:7809] | 4156 | 0.142 | -0.0509 | No |
| 59 | RABGEF1 | RAB guanine nucleotide exchange factor 1 [Source:HGNC Symbol;Acc:HGNC:17676] | 4192 | 0.139 | -0.0519 | No |
| 60 | EPS8 | epidermal growth factor receptor pathway substrate 8 [Source:HGNC Symbol;Acc:HGNC:3420] | 4213 | 0.136 | -0.0517 | No |
| 61 | NOX1 | NADPH oxidase 1 [Source:HGNC Symbol;Acc:HGNC:7889] | 4438 | 0.118 | -0.0679 | No |
| 62 | NPC1L1 | NPC1 like intracellular cholesterol transporter 1 [Source:HGNC Symbol;Acc:HGNC:7898] | 4526 | 0.111 | -0.0733 | No |
| 63 | RAB7A | "RAB7A, member RAS oncogene family [Source:HGNC Symbol;Acc:HGNC:9788]" | 4533 | 0.110 | -0.0724 | No |
| 64 | DOCK4 | dedicator of cytokinesis 4 [Source:HGNC Symbol;Acc:HGNC:19192] | 4607 | 0.104 | -0.0768 | No |
| 65 | DVL1 | dishevelled segment polarity protein 1 [Source:HGNC Symbol;Acc:HGNC:3084] | 4612 | 0.104 | -0.0758 | No |
| 66 | LSM2 | "LSM2 homolog, U6 small nuclear RNA and mRNA degradation associated [Source:HGNC Symbol;Acc:HGNC:13940]" | 4641 | 0.101 | -0.0768 | No |
| 67 | GOLGA4 | golgin A4 [Source:HGNC Symbol;Acc:HGNC:4427] | 4667 | 0.099 | -0.0775 | No |
| 68 | GGA2 | "golgi associated, gamma adaptin ear containing, ARF binding protein 2 [Source:HGNC Symbol;Acc:HGNC:16064]" | 4671 | 0.098 | -0.0764 | No |
| 69 | CLTA | clathrin light chain A [Source:HGNC Symbol;Acc:HGNC:2090] | 4718 | 0.094 | -0.0789 | No |
| 70 | FARP1 | "FERM, ARH/RhoGEF and pleckstrin domain protein 1 [Source:HGNC Symbol;Acc:HGNC:3591]" | 4744 | 0.092 | -0.0797 | No |
| 71 | BICDL1 | BICD family like cargo adaptor 1 [Source:HGNC Symbol;Acc:HGNC:28095] | 4754 | 0.091 | -0.0792 | No |
| 72 | ATP7A | ATPase copper transporting alpha [Source:HGNC Symbol;Acc:HGNC:869] | 4814 | 0.086 | -0.0828 | No |
| 73 | GCC1 | GRIP and coiled-coil domain containing 1 [Source:HGNC Symbol;Acc:HGNC:19095] | 5010 | 0.072 | -0.0973 | No |
| 74 | TMEM127 | transmembrane protein 127 [Source:HGNC Symbol;Acc:HGNC:26038] | 5173 | 0.058 | -0.1093 | No |
| 75 | TRIP11 | thyroid hormone receptor interactor 11 [Source:HGNC Symbol;Acc:HGNC:12305] | 5233 | 0.054 | -0.1133 | No |
| 76 | OPTN | optineurin [Source:HGNC Symbol;Acc:HGNC:17142] | 5323 | 0.046 | -0.1197 | No |
| 77 | RAB11FIP2 | RAB11 family interacting protein 2 [Source:HGNC Symbol;Acc:HGNC:29152] | 5388 | 0.041 | -0.1243 | No |
| 78 | SH3BP4 | SH3 domain binding protein 4 [Source:HGNC Symbol;Acc:HGNC:10826] | 5470 | 0.035 | -0.1302 | No |
| 79 | BNIP3 | BCL2 interacting protein 3 [Source:HGNC Symbol;Acc:HGNC:1084] | 5501 | 0.032 | -0.1322 | No |
| 80 | TRIOBP | TRIO and F-actin binding protein [Source:HGNC Symbol;Acc:HGNC:17009] | 5557 | 0.028 | -0.1362 | No |
| 81 | IPO13 | importin 13 [Source:HGNC Symbol;Acc:HGNC:16853] | 5640 | 0.021 | -0.1424 | No |
| 82 | CDKL5 | cyclin dependent kinase like 5 [Source:HGNC Symbol;Acc:HGNC:11411] | 5655 | 0.019 | -0.1432 | No |
| 83 | PREB | prolactin regulatory element binding [Source:HGNC Symbol;Acc:HGNC:9356] | 6044 | -0.008 | -0.1738 | No |
| 84 | SORL1 | sortilin related receptor 1 [Source:HGNC Symbol;Acc:HGNC:11185] | 6143 | -0.016 | -0.1813 | No |
| 85 | ERC1 | ELKS/RAB6-interacting/CAST family member 1 [Source:HGNC Symbol;Acc:HGNC:17072] | 6147 | -0.016 | -0.1814 | No |
| 86 | RAF1 | "Raf-1 proto-oncogene, serine/threonine kinase [Source:HGNC Symbol;Acc:HGNC:9829]" | 6187 | -0.019 | -0.1842 | No |
| 87 | RRAGC | Ras related GTP binding C [Source:HGNC Symbol;Acc:HGNC:19902] | 6202 | -0.021 | -0.1850 | No |
| 88 | AMBRA1 | autophagy and beclin 1 regulator 1 [Source:HGNC Symbol;Acc:HGNC:25990] | 6213 | -0.022 | -0.1855 | No |
| 89 | TBC1D30 | TBC1 domain family member 30 [Source:HGNC Symbol;Acc:HGNC:29164] | 6231 | -0.023 | -0.1866 | No |
| 90 | GDI2 | GDP dissociation inhibitor 2 [Source:HGNC Symbol;Acc:HGNC:4227] | 6525 | -0.044 | -0.2092 | No |
| 91 | NUTF2 | nuclear transport factor 2 [Source:HGNC Symbol;Acc:HGNC:13722] | 6536 | -0.045 | -0.2094 | No |
| 92 | RIN2 | Ras and Rab interactor 2 [Source:HGNC Symbol;Acc:HGNC:18750] | 6538 | -0.045 | -0.2089 | No |
| 93 | RAP1A | "RAP1A, member of RAS oncogene family [Source:HGNC Symbol;Acc:HGNC:9855]" | 6580 | -0.048 | -0.2115 | No |
| 94 | RPH3A | rabphilin 3A [Source:HGNC Symbol;Acc:HGNC:17056] | 6625 | -0.052 | -0.2143 | No |
| 95 | DMXL2 | Dmx like 2 [Source:HGNC Symbol;Acc:HGNC:2938] | 6655 | -0.053 | -0.2159 | No |
| 96 | WAS | WASP actin nucleation promoting factor [Source:HGNC Symbol;Acc:HGNC:12731] | 6666 | -0.054 | -0.2161 | No |
| 97 | RIN1 | Ras and Rab interactor 1 [Source:HGNC Symbol;Acc:HGNC:18749] | 6730 | -0.058 | -0.2203 | No |
| 98 | ANKRD27 | ankyrin repeat domain 27 [Source:HGNC Symbol;Acc:HGNC:25310] | 6770 | -0.061 | -0.2226 | No |
| 99 | EHD1 | EH domain containing 1 [Source:HGNC Symbol;Acc:HGNC:3242] | 6846 | -0.067 | -0.2277 | No |
| 100 | NCF2 | neutrophil cytosolic factor 2 [Source:HGNC Symbol;Acc:HGNC:7661] | 6871 | -0.069 | -0.2287 | No |
| 101 | TSC2 | TSC complex subunit 2 [Source:HGNC Symbol;Acc:HGNC:12363] | 6946 | -0.075 | -0.2336 | No |
| 102 | IPO5 | importin 5 [Source:HGNC Symbol;Acc:HGNC:6402] | 6949 | -0.076 | -0.2328 | No |
| 103 | DIAPH1 | diaphanous related formin 1 [Source:HGNC Symbol;Acc:HGNC:2876] | 7072 | -0.086 | -0.2413 | No |
| 104 | AFDN | "afadin, adherens junction formation factor [Source:HGNC Symbol;Acc:HGNC:7137]" | 7077 | -0.086 | -0.2405 | No |
| 105 | LCP1 | lymphocyte cytosolic protein 1 [Source:HGNC Symbol;Acc:HGNC:6528] | 7085 | -0.086 | -0.2400 | No |
| 106 | RIN3 | Ras and Rab interactor 3 [Source:HGNC Symbol;Acc:HGNC:18751] | 7110 | -0.089 | -0.2407 | No |
| 107 | RANBP17 | RAN binding protein 17 [Source:HGNC Symbol;Acc:HGNC:14428] | 7194 | -0.096 | -0.2461 | No |
| 108 | CAV1 | caveolin 1 [Source:HGNC Symbol;Acc:HGNC:1527] | 7205 | -0.097 | -0.2456 | No |
| 109 | MTSS2 | MTSS I-BAR domain containing 2 [Source:HGNC Symbol;Acc:HGNC:25094] | 7240 | -0.098 | -0.2470 | No |
| 110 | TBC1D13 | TBC1 domain family member 13 [Source:HGNC Symbol;Acc:HGNC:25571] | 7261 | -0.100 | -0.2473 | No |
| 111 | GCC2 | GRIP and coiled-coil domain containing 2 [Source:HGNC Symbol;Acc:HGNC:23218] | 7280 | -0.101 | -0.2475 | No |
| 112 | OCRL | OCRL inositol polyphosphate-5-phosphatase [Source:HGNC Symbol;Acc:HGNC:8108] | 7319 | -0.103 | -0.2492 | No |
| 113 | EVI5 | ecotropic viral integration site 5 [Source:HGNC Symbol;Acc:HGNC:3501] | 7616 | -0.128 | -0.2709 | No |
| 114 | AIMP1 | aminoacyl tRNA synthetase complex interacting multifunctional protein 1 [Source:HGNC Symbol;Acc:HGNC:10648] | 7638 | -0.130 | -0.2709 | No |
| 115 | PAK2 | p21 (RAC1) activated kinase 2 [Source:HGNC Symbol;Acc:HGNC:8591] | 7762 | -0.139 | -0.2788 | No |
| 116 | KNTC1 | kinetochore associated 1 [Source:HGNC Symbol;Acc:HGNC:17255] | 7779 | -0.140 | -0.2783 | No |
| 117 | HPS4 | HPS4 biogenesis of lysosomal organelles complex 3 subunit 2 [Source:HGNC Symbol;Acc:HGNC:15844] | 7868 | -0.146 | -0.2834 | No |
| 118 | RAP1GAP | RAP1 GTPase activating protein [Source:HGNC Symbol;Acc:HGNC:9858] | 7910 | -0.148 | -0.2848 | No |
| 119 | CDC42BPB | CDC42 binding protein kinase beta [Source:HGNC Symbol;Acc:HGNC:1738] | 7943 | -0.151 | -0.2854 | No |
| 120 | RAB3GAP2 | RAB3 GTPase activating non-catalytic protein subunit 2 [Source:HGNC Symbol;Acc:HGNC:17168] | 7957 | -0.152 | -0.2844 | No |
| 121 | GGA3 | "golgi associated, gamma adaptin ear containing, ARF binding protein 3 [Source:HGNC Symbol;Acc:HGNC:17079]" | 8022 | -0.157 | -0.2875 | No |
| 122 | GGA1 | "golgi associated, gamma adaptin ear containing, ARF binding protein 1 [Source:HGNC Symbol;Acc:HGNC:17842]" | 8106 | -0.163 | -0.2920 | No |
| 123 | SRGAP2 | SLIT-ROBO Rho GTPase activating protein 2 [Source:HGNC Symbol;Acc:HGNC:19751] | 8116 | -0.164 | -0.2906 | No |
| 124 | BRAF | "B-Raf proto-oncogene, serine/threonine kinase [Source:HGNC Symbol;Acc:HGNC:1097]" | 8171 | -0.169 | -0.2927 | No |
| 125 | DAAM2 | dishevelled associated activator of morphogenesis 2 [Source:HGNC Symbol;Acc:HGNC:18143] | 8227 | -0.173 | -0.2948 | No |
| 126 | BICD2 | BICD cargo adaptor 2 [Source:HGNC Symbol;Acc:HGNC:17208] | 8253 | -0.175 | -0.2946 | No |
| 127 | PAK3 | p21 (RAC1) activated kinase 3 [Source:HGNC Symbol;Acc:HGNC:8592] | 8266 | -0.176 | -0.2932 | No |
| 128 | IQGAP1 | IQ motif containing GTPase activating protein 1 [Source:HGNC Symbol;Acc:HGNC:6110] | 8276 | -0.177 | -0.2917 | No |
| 129 | KPNB1 | karyopherin subunit beta 1 [Source:HGNC Symbol;Acc:HGNC:6400] | 8285 | -0.177 | -0.2901 | No |
| 130 | SPTBN1 | "spectrin beta, non-erythrocytic 1 [Source:HGNC Symbol;Acc:HGNC:11275]" | 8335 | -0.181 | -0.2916 | No |
| 131 | DNM1L | dynamin 1 like [Source:HGNC Symbol;Acc:HGNC:2973] | 8499 | -0.194 | -0.3020 | No |
| 132 | IQGAP2 | IQ motif containing GTPase activating protein 2 [Source:HGNC Symbol;Acc:HGNC:6111] | 8598 | -0.201 | -0.3072 | No |
| 133 | BECN1 | beclin 1 [Source:HGNC Symbol;Acc:HGNC:1034] | 8644 | -0.204 | -0.3081 | No |
| 134 | ECT2 | epithelial cell transforming 2 [Source:HGNC Symbol;Acc:HGNC:3155] | 8647 | -0.204 | -0.3057 | No |
| 135 | NDRG1 | N-myc downstream regulated 1 [Source:HGNC Symbol;Acc:HGNC:7679] | 8688 | -0.208 | -0.3062 | No |
| 136 | FGD2 | "FYVE, RhoGEF and PH domain containing 2 [Source:HGNC Symbol;Acc:HGNC:3664]" | 8696 | -0.209 | -0.3041 | No |
| 137 | DVL3 | dishevelled segment polarity protein 3 [Source:HGNC Symbol;Acc:HGNC:3087] | 8756 | -0.213 | -0.3060 | No |
| 138 | DIAPH3 | diaphanous related formin 3 [Source:HGNC Symbol;Acc:HGNC:15480] | 8758 | -0.213 | -0.3034 | No |
| 139 | PICALM | phosphatidylinositol binding clathrin assembly protein [Source:HGNC Symbol;Acc:HGNC:15514] | 8905 | -0.225 | -0.3120 | No |
| 140 | ARFIP2 | ADP ribosylation factor interacting protein 2 [Source:HGNC Symbol;Acc:HGNC:17160] | 8976 | -0.230 | -0.3146 | No |
| 141 | RAPGEF6 | Rap guanine nucleotide exchange factor 6 [Source:HGNC Symbol;Acc:HGNC:20655] | 8993 | -0.231 | -0.3129 | No |
| 142 | CSE1L | chromosome segregation 1 like [Source:HGNC Symbol;Acc:HGNC:2431] | 9000 | -0.231 | -0.3105 | No |
| 143 | ANKFY1 | ankyrin repeat and FYVE domain containing 1 [Source:HGNC Symbol;Acc:HGNC:20763] | 9061 | -0.237 | -0.3122 | No |
| 144 | POU4F1 | POU class 4 homeobox 1 [Source:HGNC Symbol;Acc:HGNC:9218] | 9300 | -0.256 | -0.3277 | No |
| 145 | ARHGAP1 | Rho GTPase activating protein 1 [Source:HGNC Symbol;Acc:HGNC:673] | 9307 | -0.256 | -0.3249 | No |
| 146 | MAP3K11 | mitogen-activated protein kinase kinase kinase 11 [Source:HGNC Symbol;Acc:HGNC:6850] | 9331 | -0.258 | -0.3234 | No |
| 147 | LZTR1 | leucine zipper like transcription regulator 1 [Source:HGNC Symbol;Acc:HGNC:6742] | 9374 | -0.262 | -0.3234 | No |
| 148 | AP1G1 | adaptor related protein complex 1 subunit gamma 1 [Source:HGNC Symbol;Acc:HGNC:555] | 9389 | -0.263 | -0.3211 | No |
| 149 | DIAPH2 | diaphanous related formin 2 [Source:HGNC Symbol;Acc:HGNC:2877] | 9402 | -0.264 | -0.3187 | No |
| 150 | XPOT | exportin for tRNA [Source:HGNC Symbol;Acc:HGNC:12826] | 9597 | -0.283 | -0.3304 | Yes |
| 151 | RASGRP3 | RAS guanyl releasing protein 3 [Source:HGNC Symbol;Acc:HGNC:14545] | 9603 | -0.284 | -0.3272 | Yes |
| 152 | ENO1 | enolase 1 [Source:HGNC Symbol;Acc:HGNC:3350] | 9606 | -0.284 | -0.3237 | Yes |
| 153 | YBX1 | Y-box binding protein 1 [Source:HGNC Symbol;Acc:HGNC:8014] | 9623 | -0.285 | -0.3213 | Yes |
| 154 | CDC42EP4 | CDC42 effector protein 4 [Source:HGNC Symbol;Acc:HGNC:17147] | 9674 | -0.289 | -0.3216 | Yes |
| 155 | PARD6A | par-6 family cell polarity regulator alpha [Source:HGNC Symbol;Acc:HGNC:15943] | 9703 | -0.292 | -0.3201 | Yes |
| 156 | DENND5A | DENN domain containing 5A [Source:HGNC Symbol;Acc:HGNC:19344] | 9714 | -0.293 | -0.3171 | Yes |
| 157 | RUSC2 | RUN and SH3 domain containing 2 [Source:HGNC Symbol;Acc:HGNC:23625] | 9756 | -0.297 | -0.3166 | Yes |
| 158 | XPO6 | exportin 6 [Source:HGNC Symbol;Acc:HGNC:19733] | 9759 | -0.297 | -0.3129 | Yes |
| 159 | RHOBTB3 | Rho related BTB domain containing 3 [Source:HGNC Symbol;Acc:HGNC:18757] | 9783 | -0.300 | -0.3109 | Yes |
| 160 | ULK1 | unc-51 like autophagy activating kinase 1 [Source:HGNC Symbol;Acc:HGNC:12558] | 9798 | -0.301 | -0.3082 | Yes |
| 161 | ABCA1 | ATP binding cassette subfamily A member 1 [Source:HGNC Symbol;Acc:HGNC:29] | 9874 | -0.309 | -0.3101 | Yes |
| 162 | RABGAP1 | RAB GTPase activating protein 1 [Source:HGNC Symbol;Acc:HGNC:17155] | 9928 | -0.315 | -0.3103 | Yes |
| 163 | RAB11FIP3 | RAB11 family interacting protein 3 [Source:HGNC Symbol;Acc:HGNC:17224] | 9961 | -0.317 | -0.3088 | Yes |
| 164 | FLNA | filamin A [Source:HGNC Symbol;Acc:HGNC:3754] | 9976 | -0.318 | -0.3058 | Yes |
| 165 | NXT1 | nuclear transport factor 2 like export factor 1 [Source:HGNC Symbol;Acc:HGNC:15913] | 9983 | -0.319 | -0.3022 | Yes |
| 166 | MAPKAP1 | MAPK associated protein 1 [Source:HGNC Symbol;Acc:HGNC:18752] | 10000 | -0.321 | -0.2994 | Yes |
| 167 | INF2 | inverted formin 2 [Source:HGNC Symbol;Acc:HGNC:23791] | 10051 | -0.326 | -0.2992 | Yes |
| 168 | FNBP1L | formin binding protein 1 like [Source:HGNC Symbol;Acc:HGNC:20851] | 10082 | -0.329 | -0.2973 | Yes |
| 169 | FMNL1 | formin like 1 [Source:HGNC Symbol;Acc:HGNC:1212] | 10090 | -0.330 | -0.2937 | Yes |
| 170 | BIN1 | bridging integrator 1 [Source:HGNC Symbol;Acc:HGNC:1052] | 10123 | -0.333 | -0.2919 | Yes |
| 171 | IARS1 | isoleucyl-tRNA synthetase 1 [Source:HGNC Symbol;Acc:HGNC:5330] | 10154 | -0.337 | -0.2900 | Yes |
| 172 | ARHGEF16 | Rho guanine nucleotide exchange factor 16 [Source:HGNC Symbol;Acc:HGNC:15515] | 10278 | -0.349 | -0.2952 | Yes |
| 173 | RANGAP1 | Ran GTPase activating protein 1 [Source:HGNC Symbol;Acc:HGNC:9854] | 10288 | -0.349 | -0.2915 | Yes |
| 174 | SGSM2 | small G protein signaling modulator 2 [Source:HGNC Symbol;Acc:HGNC:29026] | 10403 | -0.361 | -0.2959 | Yes |
| 175 | GAPVD1 | GTPase activating protein and VPS9 domains 1 [Source:HGNC Symbol;Acc:HGNC:23375] | 10410 | -0.362 | -0.2917 | Yes |
| 176 | RABGGTB | Rab geranylgeranyltransferase subunit beta [Source:HGNC Symbol;Acc:HGNC:9796] | 10431 | -0.364 | -0.2887 | Yes |
| 177 | ITPKA | inositol-trisphosphate 3-kinase A [Source:HGNC Symbol;Acc:HGNC:6178] | 10450 | -0.367 | -0.2854 | Yes |
| 178 | RAB11FIP5 | RAB11 family interacting protein 5 [Source:HGNC Symbol;Acc:HGNC:24845] | 10457 | -0.369 | -0.2812 | Yes |
| 179 | GARRE1 | granule associated Rac and RHOG effector 1 [Source:HGNC Symbol;Acc:HGNC:29016] | 10460 | -0.369 | -0.2766 | Yes |
| 180 | ATG14 | autophagy related 14 [Source:HGNC Symbol;Acc:HGNC:19962] | 10465 | -0.369 | -0.2722 | Yes |
| 181 | BIRC5 | baculoviral IAP repeat containing 5 [Source:HGNC Symbol;Acc:HGNC:593] | 10513 | -0.375 | -0.2711 | Yes |
| 182 | CYRIB | CYFIP related Rac1 interactor B [Source:HGNC Symbol;Acc:HGNC:25216] | 10612 | -0.384 | -0.2739 | Yes |
| 183 | ROCK2 | Rho associated coiled-coil containing protein kinase 2 [Source:HGNC Symbol;Acc:HGNC:10252] | 10648 | -0.388 | -0.2717 | Yes |
| 184 | IPO7 | importin 7 [Source:HGNC Symbol;Acc:HGNC:9852] | 10662 | -0.389 | -0.2678 | Yes |
| 185 | SYTL2 | synaptotagmin like 2 [Source:HGNC Symbol;Acc:HGNC:15585] | 10681 | -0.391 | -0.2642 | Yes |
| 186 | RAB11FIP1 | RAB11 family interacting protein 1 [Source:HGNC Symbol;Acc:HGNC:30265] | 10710 | -0.394 | -0.2614 | Yes |
| 187 | ARHGAP44 | Rho GTPase activating protein 44 [Source:HGNC Symbol;Acc:HGNC:29096] | 10733 | -0.397 | -0.2581 | Yes |
| 188 | PEX5 | peroxisomal biogenesis factor 5 [Source:HGNC Symbol;Acc:HGNC:9719] | 10737 | -0.398 | -0.2532 | Yes |
| 189 | PEX5L | peroxisomal biogenesis factor 5 like [Source:HGNC Symbol;Acc:HGNC:30024] | 10805 | -0.406 | -0.2533 | Yes |
| 190 | ARHGEF2 | Rho/Rac guanine nucleotide exchange factor 2 [Source:HGNC Symbol;Acc:HGNC:682] | 10808 | -0.406 | -0.2483 | Yes |
| 191 | KIF3A | kinesin family member 3A [Source:HGNC Symbol;Acc:HGNC:6319] | 10848 | -0.410 | -0.2461 | Yes |
| 192 | MYO9B | myosin IXB [Source:HGNC Symbol;Acc:HGNC:7609] | 10864 | -0.412 | -0.2421 | Yes |
| 193 | FGD1 | "FYVE, RhoGEF and PH domain containing 1 [Source:HGNC Symbol;Acc:HGNC:3663]" | 10892 | -0.416 | -0.2389 | Yes |
| 194 | MICALL2 | MICAL like 2 [Source:HGNC Symbol;Acc:HGNC:29672] | 11037 | -0.435 | -0.2447 | Yes |
| 195 | RASIP1 | Ras interacting protein 1 [Source:HGNC Symbol;Acc:HGNC:24716] | 11111 | -0.445 | -0.2448 | Yes |
| 196 | IPO4 | importin 4 [Source:HGNC Symbol;Acc:HGNC:19426] | 11119 | -0.445 | -0.2396 | Yes |
| 197 | NCKAP1 | NCK associated protein 1 [Source:HGNC Symbol;Acc:HGNC:7666] | 11256 | -0.465 | -0.2444 | Yes |
| 198 | MYCBP2 | MYC binding protein 2 [Source:HGNC Symbol;Acc:HGNC:23386] | 11377 | -0.483 | -0.2477 | Yes |
| 199 | WASF1 | WASP family member 1 [Source:HGNC Symbol;Acc:HGNC:12732] | 11387 | -0.484 | -0.2423 | Yes |
| 200 | STRN3 | striatin 3 [Source:HGNC Symbol;Acc:HGNC:15720] | 11457 | -0.497 | -0.2414 | Yes |
| 201 | VPS9D1 | VPS9 domain containing 1 [Source:HGNC Symbol;Acc:HGNC:13526] | 11495 | -0.501 | -0.2379 | Yes |
| 202 | USP33 | ubiquitin specific peptidase 33 [Source:HGNC Symbol;Acc:HGNC:20059] | 11504 | -0.501 | -0.2321 | Yes |
| 203 | RANBP9 | RAN binding protein 9 [Source:HGNC Symbol;Acc:HGNC:13727] | 11521 | -0.504 | -0.2269 | Yes |
| 204 | KCTD13 | potassium channel tetramerization domain containing 13 [Source:HGNC Symbol;Acc:HGNC:22234] | 11552 | -0.508 | -0.2228 | Yes |
| 205 | CCDC186 | coiled-coil domain containing 186 [Source:HGNC Symbol;Acc:HGNC:24349] | 11573 | -0.511 | -0.2179 | Yes |
| 206 | MICAL3 | "microtubule associated monooxygenase, calponin and LIM domain containing 3 [Source:HGNC Symbol;Acc:HGNC:24694]" | 11638 | -0.523 | -0.2162 | Yes |
| 207 | PPP6R1 | protein phosphatase 6 regulatory subunit 1 [Source:HGNC Symbol;Acc:HGNC:29195] | 11739 | -0.539 | -0.2173 | Yes |
| 208 | CHM | CHM Rab escort protein [Source:HGNC Symbol;Acc:HGNC:1940] | 11798 | -0.549 | -0.2148 | Yes |
| 209 | ATG16L1 | autophagy related 16 like 1 [Source:HGNC Symbol;Acc:HGNC:21498] | 11799 | -0.549 | -0.2078 | Yes |
| 210 | GDI1 | GDP dissociation inhibitor 1 [Source:HGNC Symbol;Acc:HGNC:4226] | 11803 | -0.550 | -0.2010 | Yes |
| 211 | MICALL1 | MICAL like 1 [Source:HGNC Symbol;Acc:HGNC:29804] | 11912 | -0.574 | -0.2022 | Yes |
| 212 | RAPGEF4 | Rap guanine nucleotide exchange factor 4 [Source:HGNC Symbol;Acc:HGNC:16626] | 11963 | -0.586 | -0.1987 | Yes |
| 213 | CDC42SE1 | CDC42 small effector 1 [Source:HGNC Symbol;Acc:HGNC:17719] | 12018 | -0.596 | -0.1953 | Yes |
| 214 | YBX3 | Y-box binding protein 3 [Source:HGNC Symbol;Acc:HGNC:2428] | 12024 | -0.599 | -0.1881 | Yes |
| 215 | TNPO3 | transportin 3 [Source:HGNC Symbol;Acc:HGNC:17103] | 12032 | -0.600 | -0.1809 | Yes |
| 216 | ROCK1 | Rho associated coiled-coil containing protein kinase 1 [Source:HGNC Symbol;Acc:HGNC:10251] | 12046 | -0.605 | -0.1742 | Yes |
| 217 | FER | FER tyrosine kinase [Source:HGNC Symbol;Acc:HGNC:3655] | 12101 | -0.620 | -0.1706 | Yes |
| 218 | ABI2 | abl interactor 2 [Source:HGNC Symbol;Acc:HGNC:24011] | 12114 | -0.624 | -0.1636 | Yes |
| 219 | CHML | CHM like Rab escort protein [Source:HGNC Symbol;Acc:HGNC:1941] | 12132 | -0.626 | -0.1569 | Yes |
| 220 | KIF3B | kinesin family member 3B [Source:HGNC Symbol;Acc:HGNC:6320] | 12142 | -0.629 | -0.1496 | Yes |
| 221 | DAPK3 | death associated protein kinase 3 [Source:HGNC Symbol;Acc:HGNC:2676] | 12180 | -0.639 | -0.1443 | Yes |
| 222 | TNPO2 | transportin 2 [Source:HGNC Symbol;Acc:HGNC:19998] | 12227 | -0.652 | -0.1396 | Yes |
| 223 | EXPH5 | exophilin 5 [Source:HGNC Symbol;Acc:HGNC:30578] | 12230 | -0.653 | -0.1314 | Yes |
| 224 | PKN2 | protein kinase N2 [Source:HGNC Symbol;Acc:HGNC:9406] | 12237 | -0.655 | -0.1235 | Yes |
| 225 | RGP1 | "RGP1 homolog, RAB6A GEF complex partner 1 [Source:HGNC Symbol;Acc:HGNC:21965]" | 12302 | -0.677 | -0.1199 | Yes |
| 226 | MICAL1 | "microtubule associated monooxygenase, calponin and LIM domain containing 1 [Source:HGNC Symbol;Acc:HGNC:20619]" | 12483 | -0.742 | -0.1247 | Yes |
| 227 | SGSM3 | small G protein signaling modulator 3 [Source:HGNC Symbol;Acc:HGNC:25228] | 12515 | -0.752 | -0.1175 | Yes |
| 228 | BICD1 | BICD cargo adaptor 1 [Source:HGNC Symbol;Acc:HGNC:1049] | 12520 | -0.757 | -0.1082 | Yes |
| 229 | AKAP13 | A-kinase anchoring protein 13 [Source:HGNC Symbol;Acc:HGNC:371] | 12572 | -0.782 | -0.1022 | Yes |
| 230 | IPO9 | importin 9 [Source:HGNC Symbol;Acc:HGNC:19425] | 12575 | -0.783 | -0.0923 | Yes |
| 231 | GAS8 | growth arrest specific 8 [Source:HGNC Symbol;Acc:HGNC:4166] | 12598 | -0.797 | -0.0839 | Yes |
| 232 | RANBP2 | RAN binding protein 2 [Source:HGNC Symbol;Acc:HGNC:9848] | 12609 | -0.804 | -0.0744 | Yes |
| 233 | RCC1 | regulator of chromosome condensation 1 [Source:HGNC Symbol;Acc:HGNC:1913] | 12699 | -0.864 | -0.0704 | Yes |
| 234 | RIMS1 | regulating synaptic membrane exocytosis 1 [Source:HGNC Symbol;Acc:HGNC:17282] | 12704 | -0.866 | -0.0597 | Yes |
| 235 | HSP90AA1 | heat shock protein 90 alpha family class A member 1 [Source:HGNC Symbol;Acc:HGNC:5253] | 12730 | -0.893 | -0.0502 | Yes |
| 236 | CDC42EP2 | CDC42 effector protein 2 [Source:HGNC Symbol;Acc:HGNC:16263] | 12756 | -0.920 | -0.0404 | Yes |
| 237 | IPO8 | importin 8 [Source:HGNC Symbol;Acc:HGNC:9853] | 12761 | -0.924 | -0.0289 | Yes |
| 238 | ADCYAP1R1 | ADCYAP receptor type I [Source:HGNC Symbol;Acc:HGNC:242] | 12790 | -0.952 | -0.0190 | Yes |
| 239 | SIKE1 | suppressor of IKBKE 1 [Source:HGNC Symbol;Acc:HGNC:26119] | 12843 | -1.046 | -0.0097 | Yes |
| 240 | TNPO1 | transportin 1 [Source:HGNC Symbol;Acc:HGNC:6401] | 12864 | -1.108 | 0.0028 | Yes |
Table: GSEA details [plain text format]

  

Fig 2: GOMF\_GTPASE\_BINDING      
 Blue-Pink O' Gram in the Space of the Analyzed GeneSet

  

Fig 3: GOMF\_GTPASE\_BINDING: Random ES distribution      
 Gene set null distribution of ES for **GOMF\_GTPASE\_BINDING**
